# Polylactic-Co-Glycolic Acid/Alginate/Neem Oil-Reduced Graphene Oxide as a pH-Sensitive Nanocarrier for Hesperidin Drug Delivery: Antimicrobial and Acute Otitis Media Assessments

**DOI:** 10.3390/ph18030381

**Published:** 2025-03-07

**Authors:** Saeed Abdul Kareem Saeed Al-Zuhairy, Sammar Fathy Elhabal, Mohamed Fathi Mohamed Elrefai, Sandra Hababeh, Jakline Nelson, Marwa Fady, Nahla A. Elzohairy, Tassneim M. Ewedah, Ibrahim S. Mousa, Ahmed Mohsen Elsaid Hamdan

**Affiliations:** 1 Department of Pharmacy, Kut University College, Kut 52001, Wasit, Iraq; 2 Department of Pharmaceutics and Industrial Pharmacy, Faculty of Pharmacy, Modern University for Technology and Information (MTI), Mokattam, Cairo 11571, Egypt; 3 Department of Anatomy, Physiology and Biochemistry, Faculty of Medicine, The Hashemite University, Zarqa 13133, Jordan; 4 Department of Anatomy and Embryology, Faculty of Medicine, Ain Shams University, Cairo 11591, Egypt; 5 Department of Pharmaceutics, College of Pharmacy, King Saud University, Riyadh 11451, Saudi Arabia; 6 Department of Microbiology and Immunology, Faculty of Pharmacy, Nahda University, Beni-Suef (NUB), Beni-Suef 62511, Egypt; 7 Infection Control Unit, Zagazig University Hospitals, Zagazig 44519, Egypt; 8 Department of Microbiology and Immunology, Faculty of Pharmacy, Modern University for Technology and Information (MTI), Mokattam, Cairo 11571, Egypt; 9 Air Force Specialized Hospital, Cairo 19448, Egypt; 10 Pharmaceutics and Pharmaceutical Technology Department, Faculty of Pharmacy, Egyptian Russian University, Cairo 11829, Egypt; 11 Pharmaceutics Department, Faculty of Pharmacy, Sinai University, Al-Arish 45511, Egypt; 12 Department of Pharmacy Practice, Faculty of Pharmacy, University of Tabuk, Tabuk 71491, Saudi Arabia

**Keywords:** antifungal activity, anti-inflammatory, biofilm, controlled release, antimicrobial activity, stimuli-responsive hydrogels, neem oil, in silico, drug carriers

## Abstract

**Background/Objectives**: Hesperidin (HSP) is a potent phytochemical antioxidant and anti-inflammatory agent that protects against otitis media. However, due to its low solubility and bioavailability, a suitable delivery method is needed to overcome these problems. A hydrogel is a promising nanocarrier for controlled drug delivery in response to external stimuli, such as pH variations. **Methods**: Graphene oxide (GO)-based nanocarriers that encapsulate hesperidin (HSP) were further coated with a polylactic-co-glycolic acid/alginate (PLGA-Alg) hydrogel before being integrated into a green neem oil (N.O.) double emulsion to produce a synergistic effect and then characterized by different assays. **Results**: The nanocarriers exhibited a substantial particle size (168 ± 0.32 nm), with high encapsulation (89.86 ± 0.23%) and a zeta potential of 37 ± 0.43 mV. In vitro release studies conducted over 96 h indicated a sustained HSP release of 82% at pH 5.4 and 65% at pH 7.4. The GO-HSP-loaded neem oil double emulsion formulation exhibits substantial antibacterial activity, as evidenced by inhibition zones of 39 ± 0.02 mm against *Staphylococcus epidermidis*, and considerable antifungal activity against *Candida albicans*, with an inhibition zone of 43 ± 0.13 mm, along with biofilm inhibition activity. The formulation demonstrated antioxidant activity (5.21 µg/mL) and increased cell viability (90–95%) while maintaining low cytotoxicity in HSE-2 cells. A histopathological analysis confirmed that treatment with the nanocarriers reduced the levels of pro-inflammatory cytokines (IL-1β, TNF-α, TLR4, IL-6) and raised the levels of antioxidant markers (Nrf-2, SOD) in an in vivo rat model of otitis media. **Conclusions**: GO-based nanocarriers integrated into a neem oil double emulsion and coated with PLGA-Alg hydrogel deliver hesperidin with sustained release and enhanced antibacterial, antifungal, and antioxidant properties. This formulation may be used to treat otitis media and other oxidative stress diseases.

## 1. Introduction

Otitis media (O.M.) is used to describe the middle ear inflammation located posterior to the eardrum [1]. Among the several symptoms are ear pain, a sensation of fullness or pressure, hearing loss, fever, and fluid drainage [2]. External otitis, known as swimmer’s ear, is when the skin in the ear canal becomes inflamed. This inflammation can happen because of bacteria or fungi, irritation, or too much moisture. Otitis interna, also known as labyrinthitis, is swelling in the inner ear [3,4]. One could find causes for this including infections, autoimmune diseases, and other medical disorders [5]. The normal defence mechanism of the body, i.e., irritations, infections, and injuries, can all cause inflammation [6].

Hesperidin (HSP), a polyphenolic flavonoid, is found in many different plants, including tea, vegetables, olive oil, and fruits especially citrus fruits (family *Rutaceae*) [7]. Among its beneficial effects are anti-inflammatory, antioxidant, antiangiogenic, hypoglycemic, hypolipidemic, neuroprotective, chemotherapeutics, and anticancer actiony [8]. This can affect different acute and chronic skin disorders. Moreover, HSP improves the way wounds heal by affecting collagen synthesis, defence against wound infections, and bleeding prevention [9]. Multiple inflammatory pathway inhibition helps this strategy to lower inflammation and related symptoms. Among other inflammatory-related diseases, this aids in the treatment of inflammatory bowel disease, dermatitis, arthritis, and ear infections [10,11]. This facilitates the treatment of disorders linked to inflammation including ear infections, dermatitis, arthritis, and inflammatory bowel disease. Infections of various types have been effectively treated and prevented by HSP’s antimicrobial properties [12]. HSP effectively hindered the pulseless electrical activity (PEA) activity of MCR-1, resulting in a reduction in the resistance exhibited by the plasmid-encoded enzyme MCR-1 towards polymyxin EHSP, thereby disturbing the cell walls of pathogenic bacteria causing otitis and modulating metabolic processes, thus preventing their multiplication [13]. This activity can reduce the quantity of germs and the severity of infections. HSP inhibits inflammatory pathways and reduces the production of pro-inflammatory cytokines. HSP alleviates pain, swelling, and discomfort associated with otitis through the reduction in inflammation [14]. HSP enhances antibiotic efficacy by inhibiting bacterial efflux pump activity and reducing resistance. HSP is classified as a class II drug within the Biopharmaceutics Classification System (BCS) due to its low solubility in water and high permeability across biological membranes [10,15].

Neem oil (*Azadirachta indica*), extracted from the seeds of the neem tree, demonstrates antibacterial and insecticidal properties [16]. Nimbidin and nimbin found in neem oil demonstrate considerable antibacterial efficacy against *Staphylococcus aureus* and *Escherichia coli*. Nimbidin and nimbin in neem oil are effective antibacterial agents against *Staphylococcus aureus* and *E. coli* [17]. Neem oil prevents and treats skin and internal bacterial infections by inhibiting bacterial growth and reproduction [18,19]. Its ability to disrupt fungal cell membranes makes it effective against dermatophytes like *Trichophyton* and *Candida*. This treatment works for athlete’s foot, ringworm, and yeast infections. Organic gardeners love neem oil for its pest-repellent and fungicidal properties. Antibacterial neem oil treats acne by eliminating its causal bacteria and reducing inflammation [16]. Antifungals treat ringworms and athlete’s foot. Neem oil moisturizes dry, irritated skin and improves skin health. Skincare and cosmetic products often contain neem oil due to their therapeutic efficacy in treating eczema and acne [20]. Moreover, high doses of vitamin E promote scalp health and hair growth. Traditional medicine has long used neem oil to treat fevers and stomach issues. Since it scavenges free radicals, graphene oxide has also been studied for its antioxidant properties. Reactive molecules like free radicals damage cells and cause cancer and cardiovascular disease [18]. Graphene oxide, which neutralizes free radicals, has potential use in antioxidant therapies, cosmetics, and skincare products [12]. Graphene oxide has also shown promise in lowering inflammation in many different biological systems. Although it represents a healthy immune reaction, too much chronic inflammation can cause conditions including heart disease, inflammatory bowel disease, and arthritis [21]. The anti-inflammatory qualities of graphene oxide could result from its ability to control immune reactions and stop the synthesis of inflammatory molecules [22]. This fits exactly with anti-inflammatory medications and therapies. Graphene oxide (GO) is one of the most often used new transport nanocarriers because of its special properties. Through biocompatibility, graphene’s properties can transform the surface of a medicine [12]. Hydrogels are major biomaterials in biomedical applications because of their hydrophilicity, bi-compatibility, and therapeutic properties [23]. Synthetic polymers including polylactic-co-glycolic acid (PLGA) and polyvinylpyrrolidone can be used to generate them. Biocompatible, non-immunogenic, biodegradable, non-toxic carriers include polyvinyl alcohol (PVA), polyethene glycol (PEG), and natural polymers including agarose, chitosan, hyaluronic acid, alginate, gelatin, and collagen [24,25]. Because of its low toxicity, compatibility, solubility in organic solvents, complex formation ability, film-forming characteristics, and adhesive properties, all of which help to explain PLGA’s effectiveness as a polymer structure stabilizer PLGA is extensively used in a range of industries. The unique features of proteins make them perfect for use in biodegradable polymer nanoparticles [12]. This biocompatible and biodegradable material lowers toxicity, increases the efficacy of anticancer treatments, and spreads drug release in areas of inflammation. The use of graphene and graphene oxide-based carriers for oncological therapy seems appealing given the super-priority biocompatibility and stability of polymers in biological environments.

This work aimed to develop a new graphene oxide (GO)-based nanocarrier that encapsulated hesperidin (HSP), which was further coated with polylactic-co-glycolic acid/alginate (PLGA-Alg) hydrogel before being integrated into a green neem oil (N.O.) double emulsion for sustained delivery of drugs targeting ear inflammation. Neem oil was produced using a green synthesis technique; it served as a hydrophobic double emulsion to encapsulate and hide the HSP inside the oil phase. Using dynamic light scattering (DLS), zeta potential measurements, encapsulation, drug release profiles, Fourier transform infrared spectroscopy (FTIR), transmission electron microscopy (TEM), and scanning electron microscopy (SEM), the properties of the nanocomposites were investigated. Through in vitro and in vivo histological assessments and antifungal, antimicrobial, biofilm, cytotoxicity, and antioxidant activities, the efficacy of the nanocarrier system in ear inflammation treatment was investigated; an in silico assay was also investigated. An in silico study was also performed to define molecular interactions pertinent to controlling inflammation.

## 2. Results and Discussion

### 2.1. GC/MS Analysis of the Neem Oil (Azadirachta indica)

**Figure 1:**
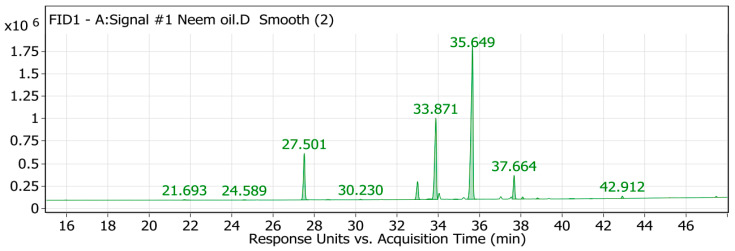
GC/MS analysis of the fixed neem oil.

**Figure 2:**
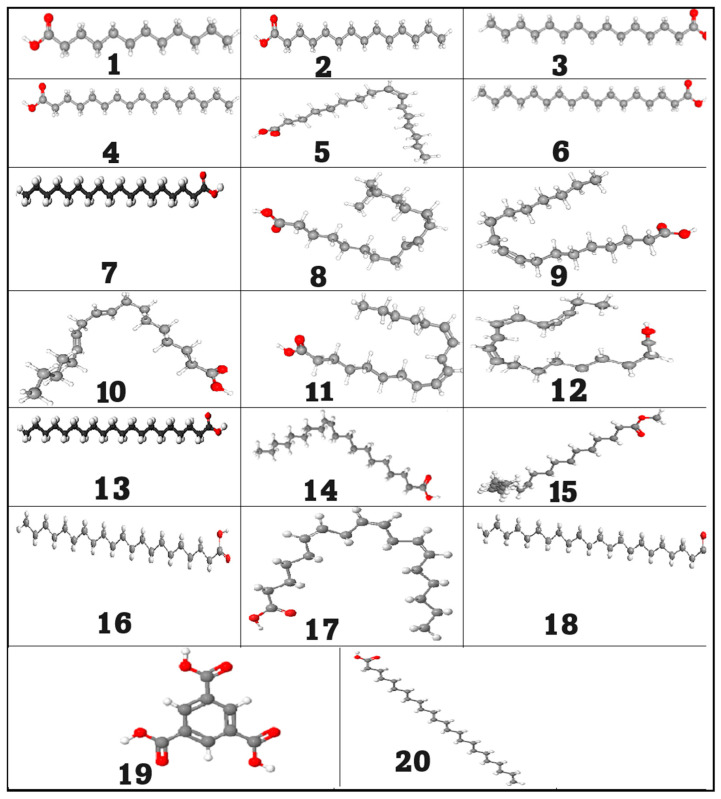
Three-dimensional compounds identified from GC/MS analysis of fixed neem oil. Note: (**1**) lauric acid, (**2**) myristic acid, (**3**) pentadecanoic acid, (**4**) palmitic acid, (**5**) palmitoleic acid, (**6**) margaric acid, (**7**) stearic acid, (**8**) elaidic acid, (**9**) oleic acid, (**10**) linolelaidic acid, (**11**) linoleic acid, (**12**) linolenic acid, (**13**) arachidic acid, (**14**) cis-11-Eicosenoic acid, (**15**) cis-11,14-eicosadienoic acid, (**16**) heneicosanoic acid, (**17**) homo-γ-linolenic acid, (**18**) behenic acid, (**19**) tricosanoic acid, and (**20**) lignoceric acid.

In the current study, the GC/MS method was used to analyze the neem oil and determine its components. A GC/MS analysis of neem fixed oil (Figure 1 and Figure 2) revealed the presence of twenty compounds, which are listed in Table 1. Neem oil contains a significant amount of palmitic acid fatty acid, as evidenced by the highest peak area observed. The total area accounted for 11.85%. Oleic acid fatty acid had the second highest peak area at 24.04% of the total area. The second highest point was located there. Oleic acid is present in a wide variety of oils and has a stellar reputation for usefulness, but with 51.18% of the total area, linoleic acid had the greatest area sum percentage of all the acids. Most scholars agree that this fatty acid is critical for good skin. Lauric acid, myristic acid, pentadecanoic acid, palmitoleic acid, margaric acid, stearic acid, elaidic acid, linolelaidic acid, linolenic acid, arachidic acid, cis-11-eicosenoic acid, cis-11,14-eicosadienoic acid, heneicosanoic acid, homo-γ-linolenic acid, behenic acid, tricosanoic acid, and lignoceric acid, as shown in Table 1 and Table S1, were also detected in diminished amounts [20,26]. Neem oil comprises fatty acids that align with prior studies regarding its composition. These fatty acids enhance the antimicrobial, anti-inflammatory, and insecticidal attributes of neem oil.

### 2.2. In Vitro Characterization of Nanoparticles

Recent research suggests graphene oxide could regenerate skin and bone; GO is ideal for these applications because electrical conductivity promotes cell adhesion, proliferation, and differentiation for tissue regeneration. GO’s antimicrobial properties may help prevent tissue regeneration infections. Graphene oxide is ideal for tissue engineering scaffolds and matrices due to its high tensile strength and flexibility. Traditional chemical exfoliation synthesizes graphene oxide (GO) by reacting graphite with strong oxidizers. This process may raise safety and biocompatibility concerns for the final GO, especially in biomedical applications. Alternative synthesis methods ensure tissue regeneration-friendly GO without harsh chemicals [12].

A DLS analysis was used to determine the particle size and the zeta potential of the nanocarrier having an average hydrodynamic diameter of 96, 148, and 168 nm for GO, PLGA-Alg-HGO, and double emulsion, respectively. This relatively small size and low polydispersity are suitable for drug delivery systems, the additional layers augmented the hydrodynamic dimensions of the nanocarriers. The zeta potentials of GO and PLGA-Alg-HGO were 25 mV and 31 mV, respectively, whereas 79% of the final nanostructures exhibited a zeta potential of 37 mV. In different layers, negative surface charges are linked to negative zeta potentials. Stable nanoparticles are indicated by absolute values greater than 30 mV [27,28]. Compared to their positively charged counterparts, negatively charged nanocomposites are more biocompatible and have a longer blood-stream circulation. The entrapment efficiency percentage and drug loading are critical criteria for evaluating the efficacy of drugs in drug delivery systems. The performance of the developed nanocarriers improves with higher drug loading [29]. In comparison to other studies, the drug loading and encapsulation efficiency of the PLGA-Alg-HGO-loaded neem oil double emulsion reached 89.86%, ranking it among the highest values reported in the literature [30,31]. As an example, the percentage discovered here is significantly higher than the drug loading of HSP-loaded polyacrylonitrile (PAN)/polyethylene oxide (PEO) electrospun nanofibers, which was determined to be 38.58 ± 6.06%. This supports the findings in Table 2 regarding the effectiveness of the nanocarrier that was created for the delivery of hesperidin.

**Figure 3:**
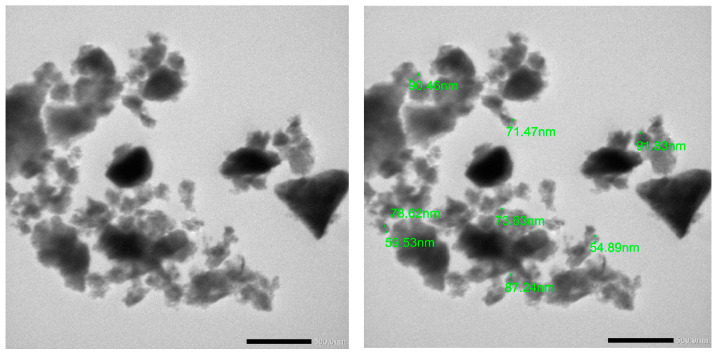
Transmission electron microscopy (TEM) image of GO.

Thorough research from the literature on antioxidants, inflammation, and biofilm prevention by the co-delivery of HSP and N.O. has been selected. The oxygen groups in graphene oxide hydroxyl, carboxyl, and epoxy most certainly are influenced by the phenolic compounds and flavonoids in the extract. This produces clusters on GO nanosheets reminiscent of nanoparticles as seen in TEM images [32], as shown in Figure 3.

**Figure 4:**
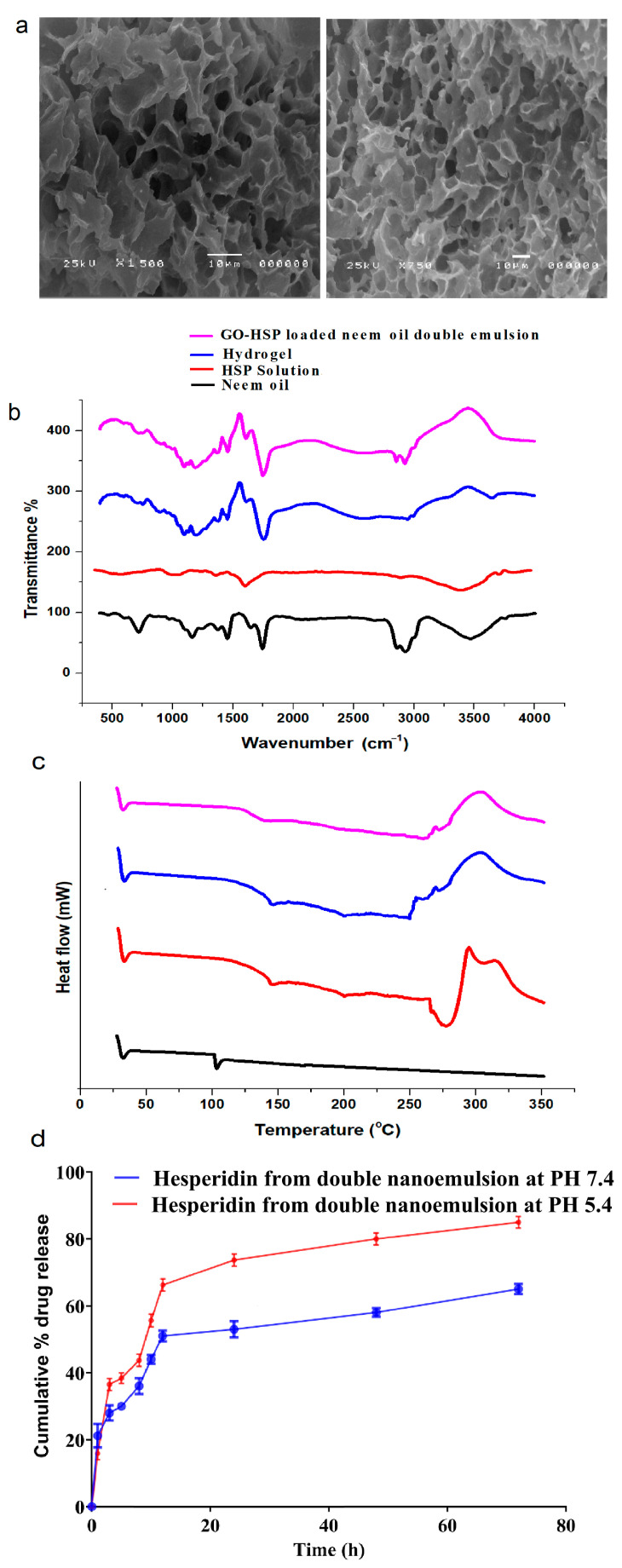
(**a**) SEM image of hydrogel, (**b**) transform infrared spectroscopy, (**c**) differential scanning calorimeter, and (**d**) hesperidin release profile from double nanoemulsion at pH 5.4 and 7.4.

The porous structure with a linked network displayed in the SEM picture of the hydrogel would help drug delivery systems. Active molecules like hesperidin are loaded and released effectively via the pores. Constantly drug diffusion and stabilization are guaranteed by the high structural integrity of the homogeneous and evenly spaced pores. A porous shape helps water absorption and swelling under specific physiological conditions, including pH sensitivity [33], thus improving the capacity of the hydrogel for controlled drug release. As shown in Figure 4a, the structural characteristics of this hydrogel position make it a good candidate for biomedical uses particularly in targeted drug delivery and sustained release systems [32].

In this study, the FTIR spectra demonstrate molecular interactions and HSP’s successful integration into the hydrogel. The FTIR spectra show molecular interactions and the successful incorporation of HSP into hydrogel and double emulsion systems. Neem oil has distinct peaks, including C-H stretching at 2920 cm^−1^ and C=O stretching at 1740 cm^−1^, indicating its fatty acid and triglyceride composition in double emulsion systems. Neem oil exhibits notable peaks at 2920 cm^−1^ and 1740 cm^−1^, which reflect its fatty acid and triglyceride composition [34]. The free HSP displays notable peaks at 3400 cm^−1^ (O-H stretching), 1650 cm^−1^ (C=O stretching of flavonoids), and 1200–1000 cm^−1^ (C-O-C and aromatic ring vibrations), which signify its flavonoid structure. In the hydrogel and double emulsion, these peaks change position and become wider [25]. For example, the O-H stretching peak at 3400 cm^−1^ becomes more noticeable, while the C=O stretching peak at 1650 cm^−1^ moves to a different position and becomes weaker. This shows that there is strong hydrogen bonding and that HSP is being enclosed. The presence of neem oil and graphene oxide peaks further confirms the stability and integration of these components [10]. These results show that hydrogel and double emulsion systems are effective in stabilizing and changing the chemical environment of HSP, which improves its functionality, as seen in Figure 4b.

Differential scanning calorimetry (DSC) was employed to investigate the thermal stability and behaviour of neem oil, hydrogel, hesperidin (HSP), and GO-HSP-loaded neem oil double emulsion systems. The free HSP exhibits melting and decompaction and clearly shows an endothermic peak at roughly 275 °C, so verifying its crystalline structure. The hydrogel shows a large temperature range; its peak is between 220 and 280 degrees Celsius. This suggests that its crystal structure is changed, which improves heat resistance through strong interactions between HSP and the hydrogel’s components (alginate-PLGA) [35,36]. The thermal peak of the GO-HSP-loaded neem oil double emulsion moves to almost 200–270 °C. This suggests combined effects and that HSP physically fits the emulsion. Peak intensity changes show that encapsulation protects HSP from heat damage, thus improving its stability [37]. As seen in Figure 4c, this enhances these formations for useful purposes.

Thermal behaviours and stability of neem oil, hesperidin (HSP), hydrogel, and GO-HSP-loaded neem oil double emulsion systems are underlined in the DSC analysis. The free HSP’s clear endothermic peak around 275 °C indicates its melting and degradation, so confirming its crystalline properties. Strong interactions between HSP and the hydrogel matrix components (alginate-PLGA) suggest disrupted crystallinity and enhanced thermal stability shown by broad thermal transitions with peaks near 220 °C and 280 °C. Likewise, the GO-HSP-loaded neem oil double emulsion shows the physical encapsulation of HSP inside the emulsion and a broadening and shift in the thermal peak to roughly 200–270 °C [15]. The observed changes and lower peak intensities suggest that encapsulation protects HSP from direct thermal degradation, so improving its stability and making these formulations more robust for use in the practical world as shown in Figure 4c.

#### 2.2.1. In Vitro Drug Release Study

The release of hesperidin from the double nanoemulsion was conducted by dialysis in two buffer media at pH 7.4 and 5.4, maintained at human body temperature (37 °C) for 80 h. As shown in Figure 4d, the release profile indicates a pH-sensitive drug delivery system.

As Figure 4d illustrates, hesperidin is released from the double nanoemulsion by dialysis in two buffer media with pH values of 7.4 and 5.4, which were kept at 37 °C, the temperature of the human body, for 72 h [37,38]. The release profile suggests a pH-sensitive drug delivery system, as shown in Figure 4d. While the total drug release after 24 h was 60% in the acidic medium (pH 5.4), in the neutral medium (pH 7.4), it was about 34%. This different release implies the integrity of the double nanoemulsion in the neutral medium, so enabling regulated drug release. The unstable structure of the nanoemulsion was weakened, and the acidic environment, which was like the environment around a tumour, made release faster [39]. The release was about 65% in the neutral environment (pH 7.4) and 90% in the acidic medium (pH 5.4) for 72 h. The acidic environment, which is like the tumour microenvironment, enabled a faster release by destabilizing the nanoemulsion structure. Over 72 h, the release rate was approximately 90% in the acidic medium (pH 5.4) and 65% in the neutral environment (pH 7.4). High pH sensitivity helps target acidic tumour tissues with the drug delivery system [37]. The oil-based middle layer of the double nanoemulsion slows a drug release and keeps it in place. The system is designed for applications that need accurate and controlled release, which is shown by its performance. The double nanoemulsion works better because of its complicated structure. It stays stable in a neutral pH environment, allowing hesperidin to be released slowly [40]. The nanoemulsion becomes less stable in acidic environments, which are common in tumour microenvironments (pH ~5.4). This speeds up the release of drugs. Making sure that the therapeutic agent reaches precisely the right place lowers the chance that it will hurt healthy tissues. A pH-sensitive nanoparticle system delivered doxorubicin. We created pH-responsive tiny particles to deliver doxorubicin. Acidic systems released more medicine, improving tumour treatment. Previous research showed pH sensitivity. In neutral conditions, a double emulsion delivery system improved drug stability and longevity, while acidic conditions allowed rapid release [31,41].

#### 2.2.2. Hesperidin Release Kinetics

**Figure 5:**
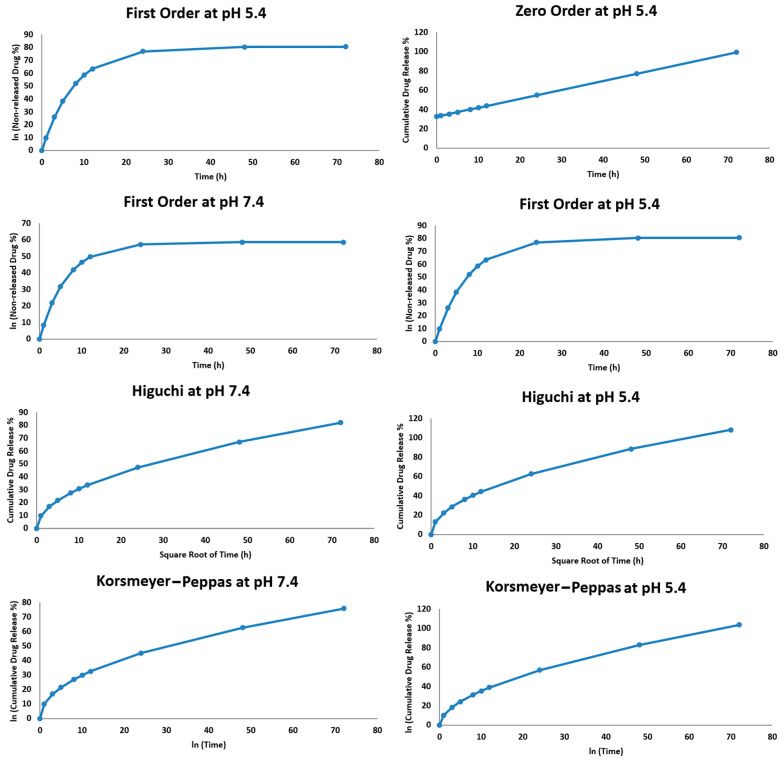
Drug release data fitted to kinetic drug release models.

The release kinetics of hesperidin from the double nanoemulsion at pH 7.4 and pH 5.4 were studied using four mathematical models: zero order, first order, Higuchi, and Korsmeyer–Peppas. Each model displays a unique drug release mechanism. The cumulative release data fit these models, and R^2^ was used to select the best-fit model. The release profile at pH 7.4 displayed a modest fit to the Higuchi model (R^2^ > 0.95), suggesting that the diffusion-dominated release mechanism is mostly used here [24]. The Korsmeyer–Peppas model produced a *p*-value of almost 0.47, suggesting that the main release mechanism is Fickian diffusion. The lower (R^2^) values of the zero-order and first-order models indicate that neither constant rate kinetics nor concentration-dependent release controls release at this pH. When the pH level was 5.4, the release profile fit better with both the Higuchi model and the Korsmeyer–Peppas model (R^2^ > 0.97). The *p*-value derived from the Korsmeyer–Peppas model at pH 5.4 was approximately 0.55, indicating anomalous (non-Fickian) diffusion, potentially encompassing both diffusion and erosion mechanisms as shown in Table 3. The destabilization of the nanoemulsion in the acidic environment, which raises the drug’s diffusion rate, is most likely the cause of the accelerated release at this pH as opposed to pH 7.4. The Higuchi model best fits the data for both pH levels and indicates that diffusion influences the mechanism of drug release. The Korsmeyer–Peppas model helps to clarify the diffusion process by demonstrating that anomalous diffusion replaces Fickian diffusion at pH 5.4 [42]. Figure 5 shows how environmental pH influences the adaptability of the nanoemulsion system and release kinetics.

### 2.3. Molecular Docking Study

The co-crystalized ligand that bound with the oleic acid binding domain (activating domain) in the PPARα target site exhibited a binding energy of −8.91 kcal/mol and formed four hydrogen bonds with Tyr464, His440, Tyr314, and Ser280, while additionally interacting with Leu321, Met355, Val332, Cys276, Ile241, Ile272, Cys275, Ile339, Met330, and His440 by thirteen hydrophobic π-Alkyl interactions. In contrast, the co-crystalized ligand complexed with the Toll-like receptor 4 target site and exhibited a binding energy of −7.02 kcal/mol. It formed two hydrogen bonds with Arg264 and interacted with Phe151, Leu149, and Ile46 through four hydrophobic π-Alkyl interactions [12,28]. The results of the docking study showed that the binding mode of palmitic acid and linoleic acid with PPARα and Toll-like receptor 4 (PDB codes: 6lx8 and 3fxi) exhibited binding energies of −8.87, −9.02, −8.75, and −8.92 kcal/mol, respectively, as shown in Table 4.

**Figure 6:**
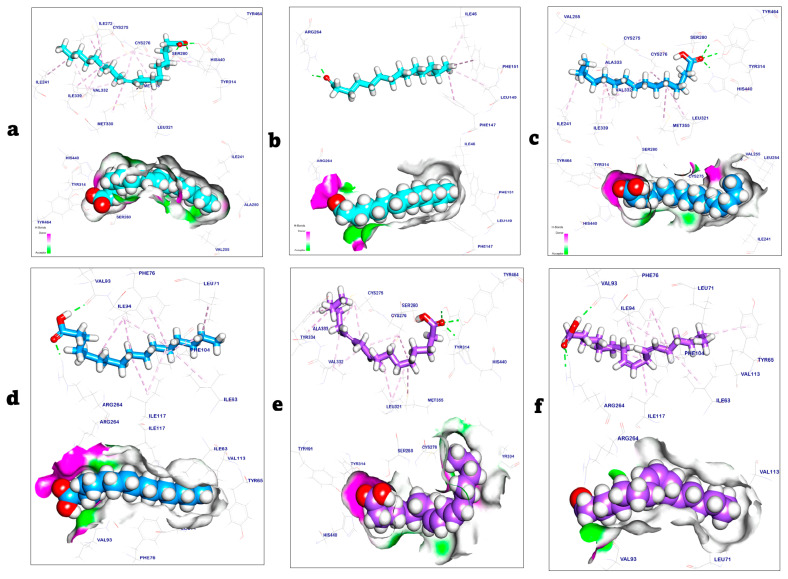
Three-dimensional view and surface mapping of the following: (**a**) co-crystalized ligand as an activator, which binds with the oleic acid binding domain in PPARα target site; (**b**) co-crystalized ligand against Toll-like receptor 4 target site; (**c**) palmitic acid as an activator, which binds with the oleic acid binding domain in PPARα target site; (**d**) palmitic acid against Toll-like receptor 4 target site; (**e**) linoleic acid as an activator, which binds with the oleic acid binding domain in PPARα target site; (**f**) linoleic acid against Toll-like receptor 4 target site.

The palmitic acid/PPARα complex showed three hydrogen bonds with Tyr464, Tyr314, and His440. Additionally, palmitic acid interacted with PPARα through eight hydrophobic π-Alkyl interactions with Ile241, Ile339, Val332, Cys275, Cys276, Met355, and Leu321. On the other hand, the palmitic acid/Toll-like receptor 4 complexes showed two hydrogen bonds with Val93 and Arg264. Moreover, palmitic acid formed eleven hydrophobic π-Alkyl interactions with Ile94, Phe76, Leu71, Ile63, Ile117, and Phe104 with the Toll-like receptor 4 target site. Linoleic acid/PPARα interacted with three hydrogen bonds with Tyr464, Tyr314, and His440. Additionally, showed thirteen hydrophobic π-Alkyl interactions with Tyr334, Ala333, Val332, Cys275, Cys276, Met355, and Leu321. On the other hand, the linoleic acid/Toll-like receptor 4 complexes showed three hydrogen bonds with Val93 and Arg264 [43,44]. Additionally, linoleic acid formed eight hydrophobic π-Alkyl interactions with Ile94, Tyr65, Phe76, Ile63, Ile117, and Phe104 with the Toll-like receptor 4 target site, as shown in Figure 6.

### 2.4. Antioxidant Activity

**Figure 7:**
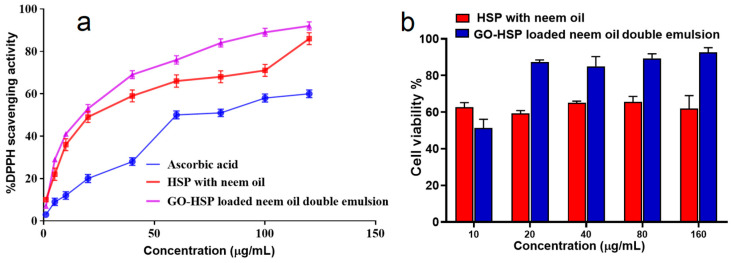
(**a**) The oxidative properties of ascorbic acid, hesperidin with neem oil, and GO-HSPloaded neem oil double emulsion against DPPH. (**b**) HSE-2 cell viability was determined after a 24 h incubation period using the MTT cell proliferation assay. Each tested cell line underwent three repetitions of the experiments.

Oxidative stress, along with other factors, causes degenerative diseases by overwhelming the body’s antioxidants, which oxidize proteins and fats. Antioxidant-rich medicinal plants improve health [45]. The antioxidant efficacy of ascorbic acid, HSP with neem oil, and GO-HSP-encapsulated neem oil double emulsion was tested using DPPH, a stable free radical that turns purple to yellow in the presence of antioxidants. Antioxidants neutralize DPPH radicals by donating hydrogen (H•), resulting in stable DPPH-H molecules. Figure 7a shows that the radical scavenging activity of ascorbic acid, HSP with neem oil, and GO-HSP-encapsulated neem oil double emulsion increased with concentration [46]. To scavenge 50% of the DPPH radical, HSP with neem oil (4.18 ± 0.43 µg/mL), GO-HSP-loaded neem oil double emulsion (5.21 ± 0.65 µg/mL), and ascorbic acid (10.67 ± 0.42 µg/mL) had IC_50_ values. The nanoemulsion has stronger antioxidant properties than ascorbic acid, which supports previous research. The phytochemicals in nanoemulsion may neutralize free radicals and thus act as an antioxidant. The results support the use of neem oil double emulsion loaded with GO-HSP as natural antioxidants to prevent degenerative diseases caused by oxidative stress [47].

Two formulations (HSP with neem oil and GO) were tested for their cytotoxic effects on HSE-2 cells at concentrations ranging from 10 to 160 µg/mL (Figure 7b). Double emulsion, HSP-loaded nitric oxide. At lower concentrations (10–40 µg/mL), both formulations show moderate cell viability, but the HSP formulation with neem oil has slightly higher viability than the GO-HSP formulation. The GO-HSP-loaded neem oil double emulsion significantly improves cell viability as concentrations increase (80–160 µg/mL), reaching 90–95% at the highest concentrations, compared to about 50% for HSP with neem oil [48,49]. The results show that the GO-HSP formulation has lower cytotoxicity, most likely due to the double emulsion system, which allows for less direct cellular interaction and the controlled release of active ingredients. Because of its increased biocompatibility, GO-HSP-loaded neem oil double emulsion is a better choice for applications requiring safe cellular interactions, such as pharmaceutical drug delivery.

### 2.5. Antimicrobial Assay

**Figure 8:**
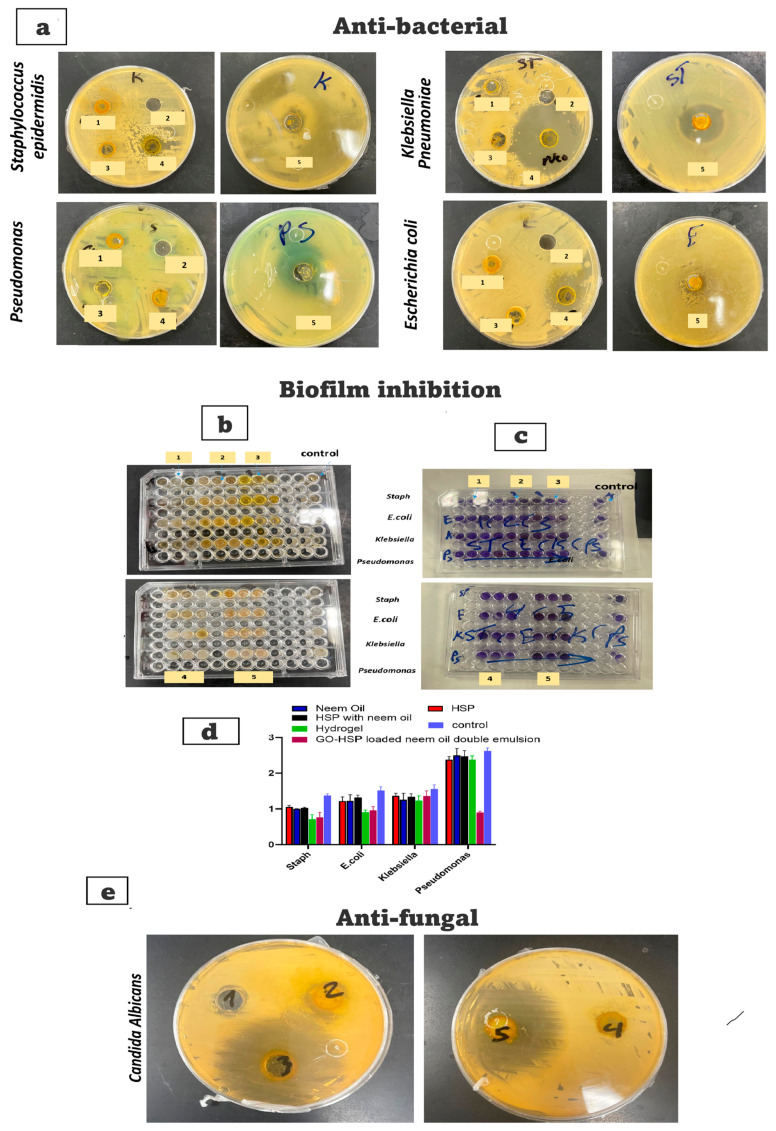
(**a**) Antibacterial activity of (1) HSP solution, (2) neem oil, (3) HSP with neem oil, (4) hydrogel, and (5) GO-HSP-loaded neem oil double emulsion against *Staphylococcus epidermidis*, *Pseudomonas*, *Klebsiella pneumoniae*, and Escherichia coli; (**b**–**d**) antibiofilm reduction and (**e**) antifungal activity against *Candida Albicans*.

HSP solution, N.O, HSP with neem oil, HSP-loaded alginate-PLGA-GO hydrogel, and GO-HSP-loaded neem oil double emulsion all showed antibacterial activity against *S. epidermidis*, *Pseudomonas*, *K. pneumoniae*, and *E. coli*. The results indicated that they had a broad-spectrum antibacterial effect against both Gram-positive and Gram-negative isolates. Figure 8a depicts the N.O. double emulsion containing GO-HSP, which has a strong antibacterial activity [46,50]. The inhibition zones for *S. epidermidis*, *Pseudomonas*, and *Klebsiella* were measured at 26 ± 0.12 mm, 28 ± 0.43 mm, and 10 ± 0.42 mm, respectively. HSP-loaded alginate-PLGA-GO hydrogel had significant antibacterial activity against *S. epidermidis* and *E. coli*, with inhibition zones of 39 ± 0.02 mm and 25 ± 0.31 mm, respectively, as shown in Figure 8a.

### 2.6. Antibiofilm Activity

The HSP Solution, neem oil, HSP with neem oil, HSP-loaded alginate-PLGA-GO hydrogel, and GO-HSP-loaded neem oil double emulsion exhibited antibiofilm properties against both Gram-positive and negative bacteria. The results state that the nanoemulsion and graphene nanoemulsion inhibited the biofilm formation [51,52]. After 24 h, the reduction percentages for *S. epidermidis* biofilm as an example of Gram-positive strain were 77% with graphene solution, 73% with Nano, 71% with graphene extract, 51% with graphene nanoemulsion, and 46% with nanoemulsion, meanwhile in case of and *E. coli*, it was 90% with HSP, 89% with N.O., 80% with HSP with neem oil, 62% with nanoemulsion, and 60% with GO-HSP-loaded neem oil double emulsion. In the case of *K. pneumoniae*, the reduction percentage was 91% with HSP, 85% with N.O., 83% with HSP with neem oil, 82% with graphene nanoemulsion, 76% with nanoemulsion, and 60% with GO-HSP-loaded neem oil double emulsion. However, *Pseudomonas* as a Gram-negative isolate was the least Gram-negative organism showing a reduction percentage was 97% with HSP, 90% with graphene solution, and nanoemulsion to 32% graphene nanoemulsion [12], as shown in Figure 8b–d.

### 2.7. Antifungal Activity Against C. albicans

Figure 8e shows antifungal activity for HSP solution, neem oil, HSP with neem oil, hydrogel, and GO-HSP-loaded neem oil double emulsion revealed significant antifungal activity against *C. albicans*. The inhibition zone of GO-HSP-loaded neem oil double emulsion was recorded at (43 ± 0.13 mm); meanwhile, the inhibition zone of graphene nanoemulsion was recorded at (37 ± 0.15 mm). The rapid increase in infectious bacterial diseases constitutes the greatest threat to human everyday life, while the emergence of multidrug resistance (MDR) has also become a very multifaceted global phenomenon. Pathogenic microorganisms develop surface attachments immediately after their adhesion, defined as biofilms, which play an indispensable role in bacterial infection due to extracellular aggregate production protecting these microorganisms from drugs and the host immune system, leading to persistent contagions [53,54]. Consequently, for fighting MDR bacteria and biofilms, the discovery of effective new antibiotics and other therapeutic agents is an especially important purpose. Graphene, discovered in 2004, is a two-dimensional carbon-based biocompatible ultra-thin nanomaterial with brilliant mechanical, electrical, and thermal properties, and its derivatives, including graphene oxide (GO) as well as reduced graphene oxide (rGO), are highly effective for hampering microbial infections. It is a renewable, low-cost, and more available nanomaterial [55]. Our investigations corroborate other most recent research that suggests nanoemulsion and graphene nanoemulsion exhibit efficient antibacterial, antibiofilm, and antifungal activities against both Gram-positive and Gram-negative bacteria and fungal infections like those triggered by *C. albicans*. To date, averting bacterial biofilm formation is of paramount approach for circumventing the spread of pathogenic microorganisms [53]. Our research findings revealed that graphene nanomaterials can be utilized as an antibiofilm. The antibiofilm activity of graphene and its derivatives is owing to their two-dimensional nanostructures with sharp edges, as well as the oxidative stress generation. Additionally, our research shows the significant antifungal activity of nano- and graphene nanoemulsion revealed against *C. albicans* which may be correlated with implant failure. The mycelium morphology of fungi is critical for biofilm formation. This high antifungal efficiency may be due to classical M1 macrophage activation for eradicating pathogens and reduced activation of M2 macrophages, decreasing the persistence of chronic infectious fungal diseases [12].

### 2.8. In Vivo Study

#### Biochemical Analysis

**Figure 9:**
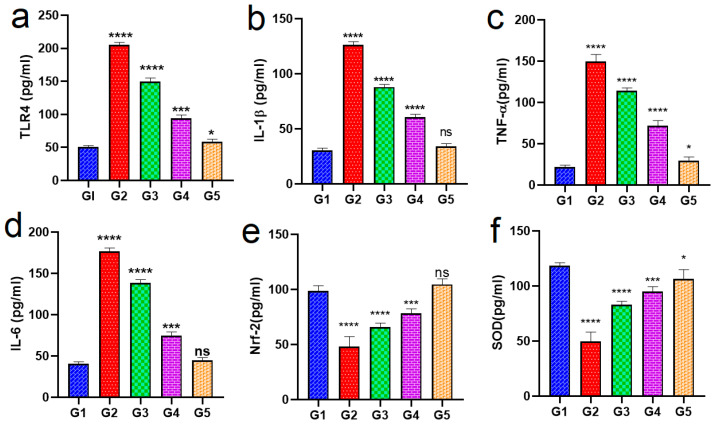
G1 is the negative control; G2 is the positive control; G3 was treated with a mixture of HSP and neem oil; G4 received HSP-loaded alginate-PLGA-GO hydrogel; and G5 received a GO-HSP-loaded neem oil double emulsion. Note: (**a**) Toll-like receptor 4 (TLR4), (**b**) Interleukin-1 beta (IL-1β), (**c**) Tumor necrosis factor-alpha (TNF-α), (**d**) Oxidative Stress Marker, (**e**) Nuclear factor erythroid 2-related factor 2 (Nrf2), and (**f**) SOD Superoxide dismutase (SOD), an antioxidant enzyme, * 0.0252, *** 0.0029, and **** <0.0001. n.s. no significant difference.

Figure 9 shows an inflammation model produced through LPS-activated macrophages. Rats with O.M. were investigated for the effects of a neem oil formula (PLGA-ALG-HsGO-HG) on inflammation using this model. The anti-inflammatory effects of neem oil, GO-HSP, and several medication combinations were assessed in this work using an ear infection model. Rats developed middle ear inflammation from PS, which raised pro-inflammatory levels (IL-1β and TNF-α) in Group 2 (positive control) relative to Group 1 (negative control). ELISA tests showed a significant inflammatory response in Group 2, with IL-1β levels of 120 ± 8 pg/mg and TNF-α levels of 150 ± 10 pg/mg. Treatment with the various formulations produced varying degrees of success in reducing inflammation. The combination of HSP and neem oil (Group 3) significantly decreased pro-inflammatory cytokine levels compared to Group 2 (*p* < 0.05). The tissue concentrations of IL-1β and TNF-α were 90 ± 10 pg/mg and 110 ± 10 pg/mg, respectively. Nonetheless, this treatment exhibited a diminished therapeutic efficacy compared to hydrogel-based formulations. Group 4 was administered HSP-loaded alginate-PLGA-GO hydrogel, resulting in a reduction in IL-1β and TNF-α levels to 60 ± 6 and 70 ± 8 pg/mg tissue, respectively (*p* < 0.001). This implies that the controlled and long-lasting release properties of hydrogel formulation greatly enhanced the decrease in inflammatory markers. Group V, which comprised the neem oil double emulsion loaded with GO-HSP showered the highest anti-inflammatory effect. Respectively, the levels of IL-1β and TNF-α were successfully calibrated at 35 ± 3 pg/mg tissue and 30 ± 3 pg/mg in comparison to the negative control group (Group 1). The amalgamation of neem oil, graphene oxide, and HSP presumably enhanced medication dispersion, targeted therapeutic effectiveness, and bio-availability [28]. The medications also affected anti-inflammatory and antioxidant markers. Group 5 demonstrated increased oxidative stress regulation, with greater levels of Nrf-2 (110 ± 10 pg/mg tissue) and SOD (130 ± 12 pg/mg tissue) relative to the other groups. The efficacy and roles of different formulations in O.M. were examined for efficacy. Natural anti-inflammatory neem oil reduced inflammation somewhat but supportively. HSP, an antioxidant and anti-inflammatory, dramatically reduced pro-inflammatory cytokines, and (GO) nanocarriers improved HSP delivery and effectiveness [27,56]. When coupled with neem oil (Group 3), HSP had moderate anti-inflammatory benefits. The Group IV HSP-loaded hydrogel exhibits high efficacy owing to its controlled and sustained drug release. The GO-HSP neem oil double emulsion (Group 5) exhibited the greatest reduction in inflammation owing to its synergistic anti-inflammatory properties. This thorough re-view demonstrates that advanced formulations such as GO-HSP neem oil double emulsion can effectively cure otitis media. These findings imply that enhanced nanocarrier systems can provide greater therapeutic efficacy than traditional drug formulations, paving the path for more effective treatment options in inflammatory disorders like otitis media [15,24].

### 2.9. Histopathology Study

**Figure 10:**
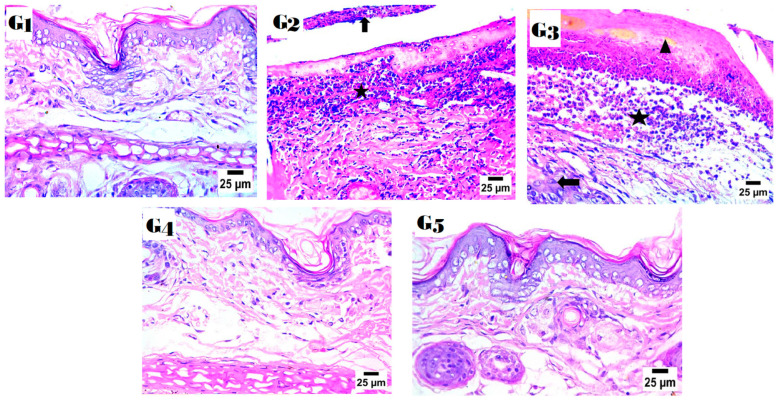
G1 is the negative control; G2 is the positive control; G3 was treated with a mixture of HSP and neem oil; G4 received HSP-loaded alginate-PLGA-GO hydrogel; and G5 received a GO-HSP-loaded neem oil double emulsion. (**G1**) Photomicrograph showing scab formation with infiltration of dermal layer by the high number of neutrophils; (**G2**) photomicrograph showing the formation of some epidermal layers (arrow) with infiltration by inflammatory cells mainly neutrophils and lymphocytes (star) covered by scab; (**G3**) photomicrograph showing moderate edema in the dermis; Note: Arrowhead (▲) appears to point to epidermal damage or necrosis, possibly indicating structural damage to the epidermis. Arrow (→) appears to highlight connective tissue alterations or inflammatory infiltration in the dermal layer, which could be due to an immune response or infection. Additionally, the star (★) marks areas of intense inflammatory infiltration (x = 25 µm). (**G4**) photomicrograph showing moderate edema in the dermis; (**G5**) photomicrograph showing the normal histological structure of epidermis and dermis (hematoxylin and eosin stain).

Figure 10 show the histological study revealed major disparities in the healing and inflammation of OM processes between the groups. Group 2 received no further treatment for seven days following the delivery of LPS and demonstrated substantial inflammatory responses, including considerable neutrophilic infiltration, which is typical of acute inflammation triggered by LPS treatment. Group 3 was given a special gel with HSP and neem oil. This resulted in much inflammation and only a little otitis healing, indicating that it was ineffective. Group IV, which received the alginate-PLGA-GO hydrogel with HSP, had less inflammation and slight swelling, indicating better inflammatory control. Group V, which recovered the best, obtained a double emulsion of GO-HSP neem oil. The return of a normal tissue structure indicated almost total healing. The anti-inflammatory and healing qualities of neem oil in concert with GO and HSP most certainly had an impact on this outcome. The results reveal that the GO-HSP neem oil double emulsion formulation is more efficient in lowering inflammation and encouraging tissue [27,57].

## 3. Materials and Methods

### 3.1. Materials and Animals

Sigma-Aldrich Chemical Co. (St. Louis, MO, USA) supplied hesperidin (HSP), PLGA (50:50, Mw: 30,000–60,000), Span 80, sodium alginate (viscous-average molecular weight: 200,000), and polylactic-co-glycolic acid; PLGA (Mw 5–15 kDa, lactic: glycolic 75:25). Neem oil was sourced from Haraz, Egypt. All solvents were obtained from VWR in Milan, Italy, and met both reagent and HPLC quality standards. Medical International Ltd. (London, UK) supplied dialysis membranes (MWCO: 3500 Da) for the in vitro release study.

**Bacterial strains**: HSP Solution, N.O., HSP with N.O., hydrogel, and GO-HSP-loaded N.O. double emulsion were tested against resistant *Staphylococcus epidermidis*, *Klebsiella pneumonia*, *Pseudomonas*, and *E. coli* from Nahda University Beni-Suef, Egypt.

**Animals**: At Cairo University’s animal house in Giza, Egypt, researchers collected forty adult male albino rats. They were divided into five groups of eight rats each, with an average age of 8 to 12 weeks and weights between 100 and 200 g. During that week, they were subjected to typical habitat conditions such as a daily light–dark cycle, a constant temperature of 22–25 °C, and a relative humidity of 45–60%. Throughout the experiment, the rats had unlimited access to water and pellet food in their plastic wire mesh cages. The Animal Ethics Committee at Cairo University’s Faculty of Pharmacy followed all animal-care protocols and procedures per Egyptian law (Approval ID: PI-3475).

### 3.2. GC/MS Analysis of the Neem Oil (Azadirachta indica)

The chemical composition of the sample was analyzed using a Trace GC-TSQ mass spectrometer (Thermo Scientific, Austin, TX, USA) equipped with a direct capillary column TG-5MS (30 m × 0.25 mm × 0.25 µm film thickness). The column oven began at 50 °C and increased at a rate of 5 °C per minute until it reached 250 °C, which was maintained for two minutes. The temperature was raised to 300 °C at a rate of 30 °C per minute for two minutes [50]. The injector and MS transfer line temperatures were set at 270 and 260 degrees Celsius, respectively. Helium was used as the carrier gas, with a steady flow rate of 1 mL/min. Diluted 1 µL samples were automatically injected using an Autosampler AS1300 connected to a gas chromatograph operating in split mode, with a 4 min solvent delay. The electron impact mass spectrometry in full scan mode at 70 eV ionization voltages resulted in mass spectra ranging from 50 to 650 *m*/*z*. The ion source temperature was set to 200 °C, and the mass spectra of the components were determined by comparing them to data from the NIST 14 and WILEY 9 databases [19].

### 3.3. Preparation of Nanocarrier

#### 3.3.1. Preparation of Graphene Oxide (GO)

**Figure 11:**
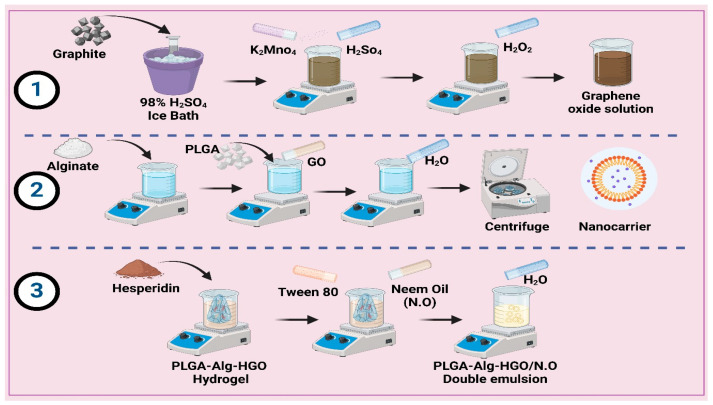
Schematic representation of polylactic-co-glycolic acid/alginate-coated graphene oxide-hesperidin and its encapsulation in a water-in-oil-in-water (W/O/W) dual emulsion containing neem oil.

First, 1 g of graphite is slowly added to 20 mL of 98% concentrated sulfuric acid (H_2_SO_4_) in an ice bath at 0 °C. Stir the mixture until the graphite is evenly distributed, adding 3 g of potassium permanganate (KMnO_4_) slowly with continuous stirring after 30 min, which causes a gradual colour change to yellowish green [12]. Next, 50 mL of distilled water is added dropwise over a period of 30 min, followed by 100 mL while stirring vigorously for 10 min [21]. To finish the oxidation process, add 35 mL of hydrogen peroxide (H_2_O_2_, 30% solution) dropwise over 30 min, resulting in foaming and a bright yellow or brownish-yellow colour. The reaction mixture sits at room temperature for 24 h. After the reaction, graphene oxide is centrifuged at 10,000 rpm for 30 min, washed with distilled water to neutral pH, and dried under a vacuum at 40 °C to powder. Complete dispersion of 50 milligrams of GO in 50 mL of distilled water is achieved by ultrasonication for 45 min during functionalization, as shown in Figure 11.

#### 3.3.2. Preparation of Polylactic-Co-Glycolic Acid/Alginate Hydrogel (PLGA-Alg-GO)

Nanocarriers coated with PLGA-Alg and GO were produced by dissolving 0.6 g of alginate in 60 mL of deionized water at room temperature, resulting in the formation of a homogeneous alginate solution. An amount of 10 mL of dichloromethane (DCM) was mixed with 0.6 g of polylactic-co-glycolic acid (PLGA) and stirred until completely dissolved. An emulsion was formed by the gradual addition of the alginate solution to the PLGA solution under vigorous stirring conditions [12]. The resulting emulsion was then sonicated in an ultrasonic bath for 10 min. Then, 60 mg of the GO nanosheets generated in the previous stage was added to the emulsion and heated and stirred until completely dispersed [58,59]. The emulsion was then introduced into 200 mL of deionized water and agitated to cause the nanocarriers to precipitate. Centrifugation was used to obtain the nanocarriers, which were then washed with deionized water and freeze-dried using a freeze-dryer, and the formulation was stored in the refrigerator at −20 °C until needed for future use. Hesperidin is hydrophobic and was dissolved in ethanol before being loaded into the hydrogel containing PLGA-Alg-coated GO [10,15]. This was followed by 30 min of stirring on the heater to obtain a homogeneous PLGA-Alg-coated GO, as shown in Figure 11.

#### 3.3.3. Preparation of the PLGA-Alg-HGO-Loaded Neem Oil Double Emulsion

A water-in-oil-in-water emulsion was prepared after the production of PLGA-Alg-GO)-encapsulated hesperidin to enable targeted and sustained release. Once the hydrogel (PLGA-Alg-HGO) had been produced in the previous step, a tween 80 surfactant with a concentration of 0.2% (*v*/*v*) was added to it. After that, while the hydrophobic phase was being heated and vigorously stirred, 5 mL of hydrogel and 10 mL of N.O. were gradually added [60]. This process created a spherical nanocarrier that contained the medicine within a hydrophobic phase. The emulsion’s hydrophobic phase, which contained 10 mL, was gradually supplemented with distilled water after 10 min [61,62]. The solution was removed from the heating stirrer and allowed to cool for 5 min after an extra 15 min; this allowed the layers to separate more easily. The drug nanoparticles were then separated from the water phase by first separating the oil phase from the sample and then centrifuging it at 6000 rpm for 10 min, as shown in Figure 10.

### 3.4. In Vitro Characterization of Nanoparticles

The particle size and surface charge of formulations were studied by using Dynamic Light Scattering (DLS) techniques and zeta potential measurements (Z.P) on a HORIBA SZ-100 Nanoparticle Analyzer (Kyoto, Japan). We dissolved and dispersed lyophilized nanocarriers PLGA-Alg-HGO (1 mg) in 1 mL of PBS to test HSP’s loading and trapping efficiency. The amount of unentrapped drug in the supernatant was determined after cooling centrifugation at 13,000 rpm for 90 min at 4 °C. The diluted supernatant’s absorbance was measured at 358 nm using a UV-VIS spectrophotometer [49,63,64]. The given Equation (1) was used to calculate the percentages of HSP loading and entrapment efficiencies.

(1) $$\mathrm{EE}\%=\frac{Total\;amount\;of\;HSP-Total\;amount\;of\;free\;HSP}{Total\;amount\;of\;HSP}\;\times \;100$$

Fourier transform infrared (FTIR) spectroscopy improves interaction. A Thermo FT-IR spectrometer (Chicago, IL, USA) recorded spectra from 400 to 4000 cm^−1^. Differential scanning calorimetry (DSC) was performed using a DSC-60 (Shimadzu Corporation, Kyoto, Japan) with indium (m.p = 156.6 °C, purity = 99.99%) at a 10 °C/min heating rate. The morphology of nanocomposites (PLGA-Alg-HGO) containing neem oil double emulsion was investigated using transmission electron microscopy (TEM) with the JEOL JEM-1230 model from Tokyo, Japan. Scanning electron microscopy (SEM) was employed to evaluated the shape of the optimized (PLGA-Alg-HGO) hydrogel, which was heated in aluminum crimped pans at 30–450 °C under nitrogen gas flow (20 mL/min) for reference, with an empty pan sealed as a control [65].

### 3.5. In Vitro Drug Release Study

By analyzing the release of medication from nanocarriers via dialysis in phosphate buffer solutions at pH 5.4 and 7.4 that were maintained in a water bath at 37 °C, the pH sensitivity of the HSP delivery method was assessed. An amount of 50 mL (*v*/*v*) of 20% ethanol phosphate buffer was added to dialysis bags with a molecular weight cutoff of 12 KDa after one millilitre of HSP-containing nanocarriers had been administered. Next, 200 μL of the sample was extracted and replaced with an equivalent volume of fresh PBS containing 20% ethanol at intervals of 0, 6, 12, 24, 48, and 72 h. At 358 nm, the amount of released HSP was measured using spectrophotometry. Plotting the cumulative percentage release of HSP over time was performed [66,67,68].

### 3.6. Hesperidin Release Kinetics

A variety of models, such as zero order, first order, Higuchi, and Korsmeyer–Peppas, were employed to analyze experimental diffusion data to investigate drug release mechanisms from nanocomposites. The optimal model was determined using the correlation coefficient (R^2^).

### 3.7. Molecular Docking Study of Neem Oil

Neem oil’s palmitic and linoleic acids were tested against PPARα, and Toll-like receptor-4 target sites to evaluate their anti-inflammatory properties. The binding sites were created with target proteins (PDB codes: 6lx8 and 3fxi) obtained from the protein data bank (https://www.rcsb.org). Initially, water molecules were extracted from the complex. The preparation options were then used to prepare and correct crystallographic defects, as well as unfilled valence atoms. The energy of the protein structure was reduced by applying CHARMM force fields. As a result, the pocket is defined and ready for docking. The 2D structures of palmitic acid and linoleic acid were created using Chem-Bio Draw 17.0 and saved as SDF files. The saved files were opened, and the three-dimensional structure was protonated and reduced using the MMFF94 force field [43,44]. The minimized structures were then prepared for docking using the Autodock vina 4.0 software’s docking feature. The receptor became rigid, but the ligands remained flexible. During the refinement process, each molecule was allowed to take twenty different positions with the proteins. The docking scores (affinity energy) of the best-fitting poses with the active sites were then recorded, and 3D views were generated using the Discovery Studio 2016 visualizer [28,43].

### 3.8. Antioxidant Activity Assay

Two millilitres of a 0.1 mM DPPH solution in methanol was mixed with two millilitres of HSP-containing neem oil and a GO-HSP-loaded neem oil double emulsion and left to sit for forty minutes. To measure antioxidant activity, positive control was used, which consisted of 50 mg/20 mL MeOH, and 0.008 mol of ascorbic acid. Absorbance was quantified via spectrophotometry within the 200–800 nm spectrum, utilizing methanol as the blank solution [69]. The amalgamation was blended and agitated at 25 °C for 45 min in an amber glass vessel. The absorbance of the generated violet hue was quantified via spectrophotometry within the range of 200 to 800 nm. The following equation was employed to determine the percentage of antioxidant activity [70,71]:

(2) $$\mathrm{Antioxidant}\;\mathrm{Activity}\;(\%)\;=\;\frac{\left(A\;control-A\;sample\right)}{A\;control}\;\times \;100$$

### 3.9. Cytotoxicity Study

The human squamous epithelial (HSE-2) cell line came from the American Type Culture Collection. Normal human dermal fibroblast cells were obtained from the American Type Culture Collection (ATCC, USA) [15]. Cells were cultured in DMEM supplemented with 10% FBS and 1% *v*/*v* penicillin-streptomycin in a humidified incubator set to 37 °C with 5% CO_2_. The MTT test was performed according to the manufacturer’s instructions (Abcam, Cambridge, MA, USA). In summary, 96-well culture plates were populated with 1 × 10^4^ cells per well, using DMEM medium with 10% FBS. The cells were incubated for 24 h at 37 °C with 5% CO_2_ in a humidified environment. The cells were then treated with varying concentrations of both plain and optimal nanoparticle formulations. After incubation, each well received 20 μL of MTT reagent and was incubated for an additional 4 h at 37 °C. The absorbance was then measured at 490 nm using a microplate ELISA reader (FLUOstar Omega, BMG, Labtech, Quakenbrück, Germany). The absorbance of the final hue corresponds directly to the number of viable cells in each sample, and the IC_50_ was calculated using GraphPad PRISM version 6 [72].

### 3.10. Antimicrobial Assay

#### 3.10.1. Antibacterial Assay and Sensitivity

Twenty millilitres of warm, melted BHA was mixed with one hundred microliters of inoculum, giving 10^7^ CFU/mL. Following this, a metal cup measuring 6 mm in diameter was used to transfer the mixture to the plate. After the BHA had hardened, the metal cups were removed, and 100 mL of plant extracts was added to the wells. At 37 °C, the plate was left to incubate for 24 h. The antibacterial activity of each formulation was evaluated by measuring the inhibitory zone diameter in millimetres. The subsequent items were incubated at 37 °C for 24 h: PLGA-Alg-HGO-loaded neem oil double emulsion, PLGA-Alg-HGO hydrogel infused with HSP and N.O., and a serially diluted GO solution. The minimum inhibitory concentration (MIC) was established by evaluating microbial turbidity and analyzing broth recovery [73]. To assess sensitivity, disc filter paper was submerged in 50 μL of each extract for 30 min and subsequently dried at room temperature. Recovery bacteria were cultivated on plates for 24 h to assess the inhibition zone [31]. The techniques employed to ascertain the minimum inhibitory concentration (MIC) and minimum bactericidal concentration (MBC) were endorsed by the Clinical and Laboratory Standards Institute. In summary, 96-well microtiter plates with a total capacity of 100 mL were employed to quantify concentrations from 0.02 to 25 mg/mL. The 10% stock solutions for each extract were subjected to two serial dilutions in brain–heart infusion broth (BHI). The wells were incubated at 37 °C with 100 mL of each tested strain at a final concentration of 1 × 10^6^ CFU/mL. The negative control consisted of 10% DMSO, the positive control comprised 0.1% (*w*/*v*) CHX, and the medium functioned as the untreated control, and antifungal activity was observed in conjunction with antibacterial activity [74].

#### 3.10.2. Antibiofilm Assay and Crystal Violet Staining (CVS) Assay

This work measured concentrations ranging from 0.02 mg/mL to 25 mg/mL using 96-well microtiter plates with a 100 mL capacity. Twice the 10% stock solution from each extract was diluted in brain–heart infusion broth (BHI). Starting at 37 °C with 100 mL of every tested strain, the wells were incubated until their final concentration was 1 × 10^6^ CFU/mL. We used 10% DMSO as the negative control, 0.1% (*w*/*v*) CHX as the positive control, and the medium as the untreated control. Each of the four strains received a 96-well microtiter plate [6,10]. The plates contained a negative control, an HSP-loaded Alg-PLGA-GO hydrogel, a GO-HSP-loaded neem oil double emulsion, and a GO solution that weakened with time. After 24 h, the wells were carefully emptied and the plates washed with a pH 7.4 phosphate buffer to remove any stuck or moving cells. After baking at 60 °C for 45 min, the plates were allowed to dry in the air. Cells that adhered were fixed by applying 150 μL of 96% methanol to each well for 15–20 min. After plate removal, adherent cells were stained with 100 μL of 0.1% crystal violet solution at room temperature for 20 min. The plates were rinsed with water at least five times to remove any remaining discolouration. The crystal violet dye was resolubilized with 150 μL of 100% ethanol, and biofilm biomass was measured semi-quantitatively. The plates’ absorbance was measured at 620 nm with a microplate reader (Epoch™ Microplate Spectrophotometer is manufactured by Agilent Technologies, Inc., located in Santa Clara, CA, USA) after careful and gentle shaking. The following equation was used to calculate the mean absorbance (OD = 620 nm) of 252 samples, with the results presented as percentage inhibition [22].

(3) $$\mathrm{Percentage}\;(\%)\;\mathrm{inhibition}\;=\;\frac{OD\;Negative\;control-OD\;Sample\;\times \;100}{OD\;Negative\;control}$$

### 3.11. In Vivo Study

#### 3.11.1. Experimental Design

This study included five groups, each with eight rats. Group 1 was the control group. The remaining rats (*n* = 32) were injected with lipopolysaccharides (LPSs). An amount of 200 μg/mL of LPSs was prepared in 0.9% normal saline and then 0.025 mL was injected into their right tympanic membranes inside their middle ear cavities using a 27-gauge needle to induce OM. The rats were randomly divided into four groups: Group (2) received no further treatment for seven days after receiving LPS; Group (3) received treatment with a mix of HSP and neem oil (0.1 mL twice daily for seven days); Group (4) received HSP-loaded alginate-PLGA-GO hydrogel; and Group (5) received a GO-HSP-loaded neem oil double emulsion (0.1 mL twice daily for seven days) [75]. All therapies began 24 h after the otitis induction. When the in vivo experiment was completed, the rats were decapitated, and the ear tissues were collected for histological and biochemical analyses in each of the assigned groups [51].

#### 3.11.2. Biochemical Analysis and Serum Preparation

All animals were anesthetized with pentobarbital sodium (200 mg/kg, IP) 48 h after the previous treatment to collect blood samples via the retroorbital sinus and scarify them with cervical dislocation. Blood samples were centrifuged, and serum was tested for TLR4, IL1β, IL-6, TNF-α, Nrf-2, and SOD (MyBioSource, San Diego, CA, USA). In a nutshell, it is a plate-based testing method developed specifically for measuring and identifying substances such as proteins, peptides, hormones, and antibodies [15,24]. The sandwich test is the most popular ELISA assay format. In this capture assay, the analyte under examination is sandwiched between the capture and detection primary antibodies, hence the name “sandwich” test. Sandwich construction is used because it is adaptable and durable. According to this technique, there is a direct correlation between the optical density and the observed biomarker levels [76].

#### 3.11.3. Histological Study

A histological analysis verified the occurrence of inflammatory responses in the middle ear. Additionally, histopathology can be utilized to identify cell infiltration, showcasing both epithelial proliferations. Autopsy samples from the sacrificed rats’ ear tissues were preserved in 10% formol saline and decalcified in formic acid for a complete day following dissection and sacrifice. After washing and fixing, dehydration was achieved through a sequence of alcohol dilutions. The paraffin-embedded dehydrated ear samples underwent heating in a hot air oven for 24 h at a temperature of 56 °C [77,78,79]. Microtome sectioning produced paraffin beeswax tissue blocks with a thickness of 4 µm. The ear tissue samples were sectioned into fragments, affixed to glass slides, stained with hematoxylin and eosin, and deparaffinized for standard examination under a light electric microscope.

### 3.12. Statistical Analysis

Experimental data were presented as mean ± SD. The statistical analyses were performed using SPSS 29.0 (IBM Co., New York, NY, USA). Shapiro–Wilk and Kolmogorov–Smirnov tests determined data normality. Parametric data were analyzed using one-way ANOVA. *p*-values under 0.05 indicated significance.

## 4. Conclusions

This study created an innovative nanocarrier system that uses graphene oxide (GO) for the targeted and pH-sensitive delivery of hesperidin (HSP). Neem oil and polylactic-co-glycolic acid/alginate hydrogel are combined to form a double emulsion containing HSP. The mixture had significant antioxidant activity, as evidenced by its IC_50_ value. The results indicated a significantly high cell viability with low cytotoxicity. In vivo experiments utilizing a rat model of otitis media demonstrated a significant reduction in the levels of pro-inflammatory cytokines TNF-α, IL-1β, TLR4, and IL-6, and greater levels of Nrf-2 and SOD in comparison to untreated controls. A histological analysis revealed that the group that received GO-HSP-loaded neem oil double emulsion had almost completely regenerated tissue. Furthermore, the formulation demonstrated strong antimicrobial activity, *S. epidermidis*, and *Candida albicans*, as well as strong antibiofilm effects. These findings show that graphene oxide, neem oil, and hesperidin can work together to create a more advanced nanocarrier system with higher therapeutic efficacy and biocompatibility. This new formulation is a promising treatment for inflammatory and infectious diseases like otitis media, and it could also be used in other pharmaceutical applications.

## Figures and Tables

**Table 1:** GC/MS analysis results for the fixed neem oil.

Peak No.	RT	Identified Compound	Area	Area Sum %	Molecular Formula	Molecular Weight	PubChem CID
4	27.5	Palmitic acid	2,777,429.22	11.85	C_16_H_32_O_2_	256.43	985
7	32.99	Stearic acid	1,165,425.88	4.97	C_18_H_36_O_2_	284.48	5281
9	33.87	Oleic acid	5,633,160.84	24.04	C_18_H_34_O_2_	282.46	445639
11	35.65	Linoleic acid	11,990,543.3	51.18	C_18_H_32_O_2_	280.43	5280450
12	37.66	Linolenic acid	1,342,567.44	5.73	C_18_H_30_O_2_	278.41	5280934
13	38.08	Arachidic acid	106,312	0.45	C_20_H_40_O_2_	312.54	10491
18	42.91	Behenic acid	114,403.13	0.49	C_22_H_44_O_2_	340.6	10492

N.B: Palmitic acid, oleic acid, and linoleic acid are compounds that have a higher area sum %.

**Table 2:** The average particle size and zeta potential measurements for PLGA-Alg-HGO-loaded neem oil double emulsion.

Formula	Particle Size (nm)	Polydispersity Index (nm)	Zeta Potential (mV)	Entrapment Efficiency (EE%)
Double emulsion	168 ± 0.32	0.21 ± 0.42	37 ± 0.43	89.86 ± 0.23

**Table 3:** Correlation coefficient of the different kinetic models for drug release.

Model	R^2^ (pH = 5.4)	R^2^ (pH = 7.4)
Zero Order	0.94	0.93
First Order	0.96	0.95
Higuchi	0.98	0.99
Korsmeyer–Peppas	0.97	0.98

**Table 4:** It shows ∆G kcal/mol of palmitic acid and linoleic acid against PPARα and Toll-like receptor 4 target site.

Targets	Tested Compounds	RMSD Value (Å)	Docking (Affinity) Score (kcal/mol)	Interactions	
				H.B	Pi Interaction
PPARα	The co-crystalized ligand	1.01	−8.91	4	13
	*Palmitic acid*	1.15	−8.87	3	8
	*Linoleic acid*	1.31	−9.02	3	13
Toll-like receptor 4	The co-crystalized ligand	1.23	−7.02	2	4
	*Palmitic acid*	1.32	−8.75	2	11
	*Linoleic acid*	1.07	−8.92	3	8

## Data Availability

The authors confirm that the data supporting the findings of this study are available within the article.

## Supplementary Materials

The following supporting information can be downloaded at: https://www.mdpi.com/article/10.3390/ph18030381/s1, Table S1: GC/MS analysis results for the fixed neem oil.

## Abbreviations

The following abbreviations are used in this manuscript:

Abbreviation	Full Form
GO	Graphene Oxide
HSP	Hesperidin
PLGA	Polylactic-Co-Glycolic Acid
Alg	Alginate
PLGA-Alg	Polylactic-Co-Glycolic Acid/Alginate
N.O.	Neem Oil
DLS	Dynamic Light Scattering
FTIR	Fourier Transform Infrared Spectroscopy
TEM	Transmission Electron Microscopy
SEM	Scanning Electron Microscopy
OM	Otitis Media
BCS	Biopharmaceutics Classification System
MIC	Minimum Inhibitory Concentration
MBC	Minimum Bactericidal Concentration
CVS	Crystal Violet Staining Assay
LPS	Lipopolysaccharides
ELISA	Enzyme-Linked Immunosorbent Assay
TNF-α	Tumour Necrosis Factor Alpha
IL-1β	Interleukin 1 Beta
IL-6	Interleukin 6
TLR4	Toll-Like Receptor 4
Nrf-2	Nuclear Factor Erythroid 2-Related Factor 2
SOD	Superoxide Dismutase
DMEM	Dulbecco’s Modified Eagle Medium
FBS	Fetal Bovine Serum
ATCC	American Type Culture Collection
BHI	Brain–Heart Infusion Broth
CHX	Chlorhexidine
DMSO	Dimethyl Sulfoxide
MTT	3-(4,5-Dimethylthiazol-2-yl)-2,5-diphenyltetrazolium Bromide
IP	Intraperitoneal
GC/MS	Gas Chromatography-Mass Spectrometry
HPLC	High-Performance Liquid Chromatography
Mw	Molecular Weight
MWCO	Molecular Weight Cut-Off
ANOVA	Analysis of Variance
PBS	Phosphate-Buffered Saline
MDPI	Multidisciplinary Digital Publishing Institute
DOAJ	Directory of Open Access Journals
TLA	Three-Letter Acronym
LD	Linear Dichroism
