# Supplementary material for: Polylactic-Co-Glycolic Acid/Alginate/Neem Oil-Reduced Graphene Oxide as a pH-Sensitive Nanocarrier for Hesperidin Drug Delivery: Antimicrobial and Acute Otitis Media Assessments
Source: Pharmaceuticals (Basel). 2025 Mar 7;18(3):381. doi: 10.3390/ph18030381 (PMC11944605; doi:10.3390/ph18030381)
Supplement: Supplementary file 1 [file pharmaceuticals-18-00381-s001.zip › pharmaceuticals-3492468-supplementary.pdf]

Table S1. GC/MS analysis results for the fixed Neem oil.

Peak no	RT	Identified Compound	Area	Area Sum %	Molecular Formula	Molecular weight	PubChem CID
1	15.93	Lauric acid	2680.63	0.01	$C_{12}H_{24}O_2$	200.32	3893
2	21.69	Myristic acid	23433.45	0.1	$C_{14}H_{28}O_2$	228.38	11005
3	24.59	Pentadecanoic acid	3991.22	0.02	$C_{15}H_{30}O_2$	242.4	12498
5	28.64	Palmitoleic acid	20596.77	0.09	$C_{16}H_{30}O_2$	254.41	5281103
6	30.23	Margaric acid	23350.38	0.1	$C_{17}H_{34}O_2$	270.45	10490
8	33.53	Elaidic acid	83760.69	0.36	$C_{18}H_{34}O_2$	282.46	5280450
10	34.84	Linolelaidic acid	23086.17	0.1	$C_{18}H_{32}O_2$	280.43	5281127
14	38.8	*cis*-11-Eicosenoic acid	50755.56	0.22	$C_{20}H_{38}O_2$	310.51	5281116
15	40.39	*cis*-11,14-Eicosadienoic acid	8447	0.04	$C_{20}H_{36}O_2$	308.48	5281098
16	40.53	Heneicosanoic acid	5423.74	0.02	$C_{21}H_{42}O_2$	326.57	5281148
17	41.4	Homo-γ-linolenic acid	1763.7	0.01	$C_{20}H_{32}O_2$	304.46	5280933
19	45.21	Tricosanoic acid	8081.08	0.03	$C_{23}H_{46}O_2$	356.64	5281162
20	47.46	Lignoceric acid	44517.84	0.19	$C_{24}H_{48}O_2$	372.68	5281150
